# Fluid pressure and flow as a cause of bone resorption

**DOI:** 10.3109/17453674.2010.504610

**Published:** 2010-07-16

**Authors:** Anna Fahlgren, Mathias P G Bostrom, Xu Yang, Lars Johansson, Ulf Edlund, Fredrik Agholme, Per Aspenberg

**Affiliations:** ^1^Division of Orthopaedics, Department of Clinical and Experimental Medicine, Linköping University, LinköpingSweden; ^2^Hospital for Special Surgery, New York, NYUSA; ^3^Division of Mechanics, Department of Management and Enineering, Linköping University, LinköpingSweden

## Abstract

**Background:**

Unstable implants in bone become surrounded by an osteolytic zone. This is seen around loose screws, for example, but may also contribute to prosthetic loosening. Previous animal studies have shown that such zones can be induced by fluctuations in fluid pressure or flow, caused by implant instability.

**Method:**

To understand the roles of pressure and flow, we describe the 3-dimensional distribution of osteolytic lesions in response to fluid pressure and flow in a previously reported rat model of aseptic loosening. 50 rats had a piston inserted in the proximal tibia, designed to produce 20 local spikes in fluid pressure of a clinically relevant magnitude (700 mmHg) twice a day. The spikes lasted for about 0.3 seconds. After 2 weeks, the pressure was measured in vivo, and the osteolytic lesions induced were studied using micro-CT scans.

**Results:**

Most bone resorption occurred at pre-existing cavities within the bone in the periphery around the pressurized region, and not under the piston. This region is likely to have a higher fluid flow and less pressure than the area just beneath the piston. The velocity of fluid flow was estimated to be very high (roughly 20 mm/s).

**Interpretation:**

The localization of the resorptive lesions suggests that high-velocity fluid flow is important for bone resorption induced by instability.

## Introduction

Unstable implants in bone become surrounded by an osteolytic zone. This has been described for insufficiently fixed screws in classical work by Perren et al. (2002). The pathophysiological background of this phenomenon has not been investigated. Most work on this subject is related to joint replacements. Mechanical factors related to the operative procedure and early postoperative processes contribute to implant loosening (Mjoberg 1997, Greenfield and Bechtold 2008). Insufficient initial fixation or early loss of fixation lead to prosthetic instability, thereby causing pressure fluctuations at the interface tissue during loading (Manley et al. 2002). Microinstability is related to migration of joint implants, as measured by radiostereometric analysis (RSA). Furthermore, migration is predictive of later mechanical loosening (Karrholm et al. 1994, Ryd et al. 1995). Early prosthetic migration is related to an osteoclastic response to the surgical trauma. This is demonstrated by the observation that the migration of knee prostheses can be reduced by bisphosphonates (Hilding et al. 2000, Hilding and Aspenberg 2006, 2007).

Prosthetic migration appears to require a fibrous tissue at the interface between prosthesis and bone. This will form as a consequence of the lack of initial stability (De Man et al. 2005, Lioubavina-Hack et al. 2006). Several animal models have demonstrated that micromotion of implants is associated with local bone resorption and formation of fibrous tissue (Aspenberg and Herbertsson 1996, Perren 2002, Lioubavina-Hack et al. 2006). Compression of the fibrous interface tissue may also generate fluid pressure and fluid flow, leading to bone loss (Skripitz and Aspenberg 2000, De Man et al. 2005).

It is a generally accepted hypothesis that fluid flow within the bone matrix plays a key role in a strain-sensing mechanism involved in the normal adaptive response of bone (Van der Vis et al. 1998a, b, Skripitz and Aspenberg 2000, Qin et al. 2002, Astrand et al. 2003). Bone strain in vivo leads to pressure gradients and fluid flow through osteocyte canaliculi. This is sensed by the osteocytes, which signal to cells at the bone surface to increase bone formation (Cowin 2002, Burger et al. 2003, Qin et al. 2003, Tan et al. 2007). In this paper we analyze the possibility of a different mechanism in which fluid flow leads to osteolysis, possibly because it occurs outside the bone or because it has a different magnitude.

The fluid pressure in the pseudo-joint of a loose hip prosthesis is elevated at rest, and ranges from 3 to 280 mmHg depending on the position (Robertsson et al. 1997). During different mechanical maneuvers in patients with hip prostheses, such as walking and rising from a chair, the pressure can be 155–776 mmHg (Hendrix et al. 1983). Thus, fluid pressure and flow can also be induced in the absence of microinstability. This could contribute to the formation of osteolytic cavities that can also be observed around ossseointegrated implants (Harris et al. 1976, Anthony et al. 1990, Walter et al. 2004).

Pressure-induced bone resorption has been studied in several animal models (Skripitz and Aspenberg 2000, Astrand et al. 2003). In the present study, we used a model with only 2 min of intermittent fluid pressure applied twice daily (Skripitz and Aspenberg 2000). A piston was moved perpendicularly to an isolated bone surface at the proximal rat tibia, thereby creating fluid pressure and flow. When this stimulus was applied, the bone adjacent to the piston was largely resorbed after 5 days. The strong resorptive response in this model has been reported previously (Skripitz and Aspenberg 2000). The goal of the present study was to quantify the pressure and flow behind this response, and to describe the 3-dimensional distribution of the osteolytic lesions, in order to elucidate the roles of pressure and flow.

## Material and methods

### Animal model

**Figure 1.**
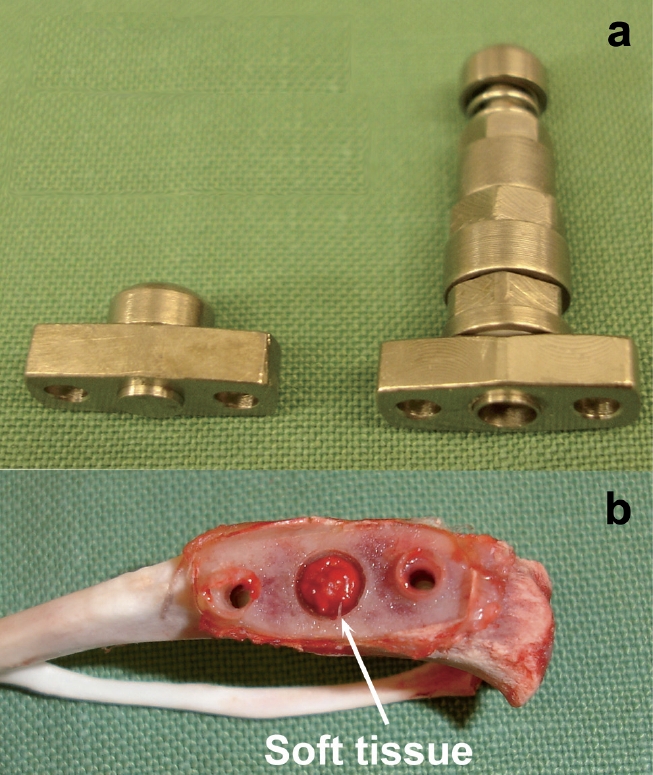
a) The plate with the central plug (left) and the pressure piston (right) b) tibia showing the bone surface under the titanium plate and the newly formed soft tissue under the pressure piston.

**Figure 2.**
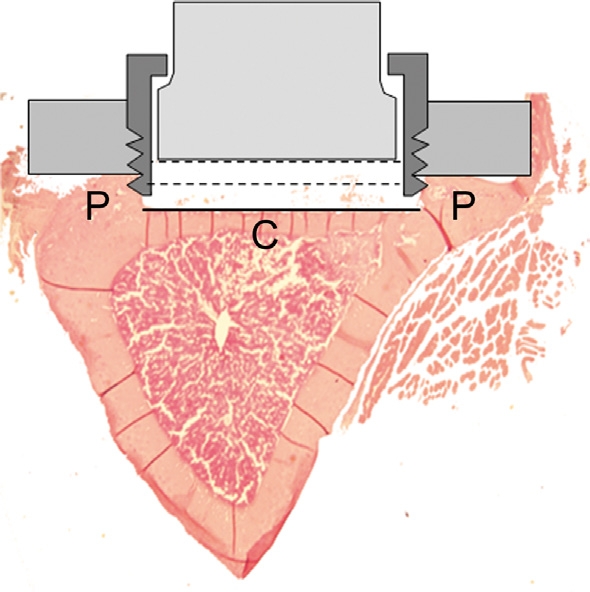
Transverse section of proximal tibia. The original plug protruded 0.1 mm deeper than the piston (the black line). The lowest position for the piston is 0.6 mm from the bone surface (the middle dotted line). The highest position is 1.4 mm from the bone surface, (the upper dotted line). Thus, a total volume of 8.6 mm^3^ under the piston, reduced to 4.5 mm^3^ when the piston is moved to the lower position during a pressure cycle.

74 rats (mean weight 406 g (SD 21), and 11 weeks old when operated) were used in an animal model of pressure-induced prosthesis loosening. The model is a modified and improved version of the model used by Skripitz and Aspenberg (2000). The present model uses a more accurate pressure piston, with silicon details that prevent leakage, and a standardized spring preload of 4 N (Figure 1a). In a first operation, a titanium plate with a center plug was fastened with 2 cortical screws at the medial aspect of the rat proximal tibia. The center plug protruded 0.5 mm into a milled depression in the bone cortex. New bone was allowed to grow up to the titanium surface of the central plug to form a flat bone surface (Figure 1b). In a second operation, when the titanium plate and central plug were osseointegrated, the plug was replaced with a pressure piston. The pressure piston was sealed with a silicon tube and an O-ring. With the piston in place, a 1.4-mm distance is left for soft tissue to form between the piston and the flat bone surface (Figure 2). When loaded, the soft tissue is compressed by the piston, thereby creating a fluid pressure, propagated down onto the underlying bone. The piston does not reach the bone, but stops 0.6 mm from the surface of the bone, thereby avoiding direct contact. After being pressed down by an external force, the piston is moved up by the internal spring.

During the experiments, the piston was subjected to a transcutaneous force of 8 N by a manually operated dynamometer applied to the skin over the piston, forcing it downwards and compressing the soft tissue. The magnitude of pressure generated depends on the resistance to fluid flow within the tissue and the speed at which the piston moves. The velocity of the piston was approximately 14 mm/s, calculated from a distance of the piston of 0.7 mm and a time of 0.05 seconds. The time was quantified by counting the numbers of frames of the piston moving from the lowest to the highest position in a 25-Hz camcorder (data not shown). The pressurized bone area was 6.2 mm^2^. Thus, in the unlikely case that the fluid flow had been zero, the pressure would have been approximately 0.6 MPa (4,560 mmHg). Each episode of pressure comprised 20 pressure cycles during 2 min at 0.17 Hz, applied twice a day.

The day after the last pressure episode, the animals were killed and the tibia was cut at the level of the proximal and distal screw hole for further analysis. These experiments were carried out within the context of institutional guidelines for care and treatment of experimental animals, and after approval by Linköping ethical committee (date of issue: March 30, 2004; registration number: 33-04; and date of issue: November 21, 2006; registration number: 63-06)

### Surgery

The rats were anesthetized with 5% isoflurane gas. They received a preoperative subcutaneous injection of 25 mg/kg tetracycline and 5 mg/kg carprofen at the first operation. At the second operation, they received 0.015 mg/kg buprenorphine instead of carprofen, to avoid any effect of NSAID on bone metabolism. Under aseptic conditions, a 5–6 mm longitudinal incision was made along the tibia. The periosteum was reflected proximally to the physis. A depression in the tibial cortex was milled out to correspond to the pressure area of the middle hole. After the depression was prepared, the titanium plate was screwed onto the predominantly cortical bone.

### Evaluation of morphology and in vivo fluid pressure

50 rats were randomized to 5 groups, to study the osteolytic process. They were killed after 0 (n = 10), 5 (n = 20), and 14 days (n = 20). At 5 and 14 days, 10 animals in each group were non-pressurized controls. Half of the animals in the latter two groups did not undergo pressure treatment (controls). The animals were killed and the bone specimen under the titanium plate was placed in formalin for further processing.

### In vivo fluid pressure measurement

**Figure 3.**
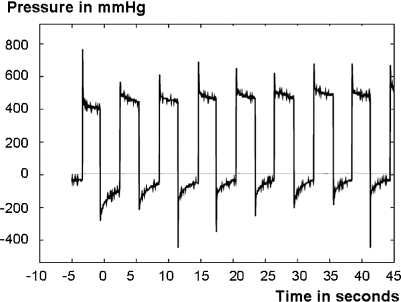
A typical pressure curve showing the maximum fluid pressure spikes and the following fluid pressure plateaus.

Immediately before the rats were killed, the piston loading procedure described above was repeated with a pressure transducer inserted between the piston and the plate, in order to measure the pressure levels produced by the piston. Honeywell pressure transducers from the 26PC series with nominal ranges from 0–50 mmHg to 0–1,550 mmHg above atmospheric pressure were used. The fluid pressure under the piston was measured using the same loading protocol as previously during the experiment. Also, in one experimental series, the pressure in the bone marrow was measured with a second pressure transducer of the same type, using a coupling screwed into the bone marrow from a site opposite the titanium plate. The thread of the coupling was sealed with silicon mass. The transducers were connected to Vishay Measurements Group 2210 amplifiers and the pressure curves were saved to disc using an Agilent 54624A oscilloscope. In the experiments, we observed that the first or first few cycles were usually a little different, with a higher pressure. Thus, to compute representative values, averages of the third through seventh cycles were used in all calculations. We also noted that the pressure cycles usually had an initial spike of short duration and then settled to a slowly decaying or constant value (Figure 3). In an attempt to quantify the typical duration of these spikes, the time for the pressure to decay from its maximum value to a value three-quarters down between the maximum and average pressure values was calculated. Here, the third through seventh cycles were used for all the specimens, except the specimens used for intramedullary fluid pressure measurements, which were excluded completely.

Two types of pressure values were collected. The maximum pressure is defined as the maximum pressure during that part of the cycle when the piston is compressed, relative to atmospheric pressure. The plateau pressure is the average pressure during the first half of the cycle when the piston is compressed, relative to the average pressure during the second half of the cycle when the piston is released before the next cycle. When calculating the plateau pressure, the first 0.5 s of each pressure episode was excluded in order to exclude the initial spike from this value; this correction did not, however, change the results in any major way.

### Microcomputer tomography (CT scanning) and morphometry

The experimental area under the piston was scanned using the MS-8 Small Animal Microcomputed Tomography System (GE Healthcare, Ontario, Canada) with a resolution of 23 μm. The tibiae were placed in a saline-filled tube and scanned at 80 kV and 80 mA. 400 exposures were taken for each scan. Threshold selection and morphometric analysis were performed with the MicroView 2.1 computer program supplied by GE Healthcare. The region of interest was a 4-mm diameter cylinder, 0.8 mm deep. The center of the cylinder was placed where the center of the piston had been. Bone volume, bone volume fraction (BVF), and bone mineral density (BMD) were measured for each scan using a threshold of 1,667 Hounsfield units. 3-D morphometric pictures were used for descriptive morphometry.

### Fluid flow velocity

The fluid flow velocity through the soft tissue is defined here as the fluid volume per second pushed by the piston divided by that part of the cross-sectional area that is soft tissue, excluding bone area. To find the area to be used in this calculation, it was assumed that the ratio of bone area to total area is the same as the bone volume fraction (BVF) from the micro-CT scan of the specimen. The bone area is then the total area multiplied by BVF, while the soft tissue area we seek is the remainder. In this way, the soft tissue velocity was calculated as piston velocity / (1 – BVF).

### Morphology of CT scans

To be able to identify typical patterns more easily, we created images showing the average osteolysis levels for several specimens. To do this, 2 equivalent points were identified on each image and the images were rotated, translated, and stretched to make these points coincide. On the frontal views presented, these points were the center points of the holes for the fastening screws, with the sections used selected to be as close as possible to the bone layer formed adjacent to the originally inserted central plug. The numerical grayscale values, normalized by inspection so that a value of 1 (maximum porosity) represented a cavity and a value of 0 (no porosity) represented solid bone, were then simply added to create an aggregated gray scale. Also, to have as much data as possible going into the averages, we used symmetry reflections. In the frontal views, 9 or 10 specimens were used with one symmetry defined by the line through the center of the screw-holes and one symmetry at right angles to this line, so that each point in the picture represented 36–40 data points. No specimens were excluded because of being atypical in the amount of osteolysis, but a few of the individual pictures were excluded because of the difficulty in some cases in identifying the points used to center the images. Finally, to make the images easier to understand, the grayscales were replaced by a color scale, with the darkest magenta representing solid bone and yellow representing very little bone, and with white representing the extreme values of little or no bone. Adding white at the end of the color scale serves the purpose of making the background color white, so that the contour of the specimens stands out from the background.

### Histology and immunohistochemistry

The specimens were decalcified in formic acid and embedded in paraffin, and processed for histological and immunohistochemical evaluation. Hematoxylin and eosin staining was used for desciptive histology. Standard immunoperoxidase protocols using polyclonal anti-mouse cathepsin K antiserum (Zenger 2007) were used to visualize bone-resorbing osteoclasts. Histology and immunohistochemistry were assessed under light microscopy to describe the osteolytic process.

### Model characteristics

To understand and describe the phenomena observed in the animal model more carefully, we performed 4 separate experiments that examined the characteristics of the model (Table).

**Table:** Study design of experiments describing characteristics of the mode

Experiment	n	Follow-up (days)	Methods
Intramedullary fluid pressure	4	0	Fluid pressure
Fluid displacement	6	0	Light microscopy
Motion-controlled loading	4	0	Materials testing machine
Impact loading	4	5	Micro-CT
			Light microscopy
			Immunohistochemistry
Trauma versus pressure	6	5	Light microscopy
			Immunohistochemistry

### Intramedullary fluid pressure

Measurements of fluid pressure below the piston showed only a minor decrease from the start of the experiments to the later time points with major osteolysis. To assess whether this pressure extended through the bone under the piston into the bone marrow, we performed an additional experiment in which the fluid pressure was measured in the bone marrow cavity in 4 rats. 5 days after insertion of the piston, when loading was normally to be started, the marrow pressure was measured before and after a 1.2-mm diameter hole was drilled through the bone under the piston, into the marrow cavity.

### Fluid displacement

To investigate the motion of fluid after leaving the space below the piston into the bone tissue, 6 controls rats were used. 5 days after piston insertion, when the loading phase would normally start, the piston was temporarily removed, titanium particles (Alfa Aesar, Karlsruhe, Germany) were applied to the soft tissue below it, and the piston was replaced (90% of the titanium particles were smaller than 3.6 μm). The piston was pressurized cyclically as previously described, but for 10 min. Immediately afterwards, the rats were killed and the bone specimens frozen. Thin slices of 0.5 mm were sawed with a bone saw and analyzed under light microscopy at 10× magnification to determine the distribution of the titanium particles, as an indication of the direction of fluid flow.

### Motion-controlled loading

To understand the events during the pressure spike during the first few tenths of a second, the experimental conditions were replicated approximately using a displacement-controlled materials testing machine. 5 days after piston insertion, 4 rats were subjected to motion-controlled loading of the piston using the materials testing machine (100 R; DDL Inc., Eden Praire, MN). After anesthesia, and with the skin opened, the piston in the rat was exchanged for a specially prepared piston that could be fixated in a custom-made experimental set-up. The piston and rat leg were firmly attached to the experimental set-up, allowing the materials testing machine to push the piston in a controlled manner, simulating the manual treatment otherwise used. The machine moved the piston downward with a velocity 20 mm/s for 0.3 mm, then halted for 3 s, and then moved 0.3 mm in the opposite direction at the same speed. This cycle was repeated 3 times. The whole procedure was then repeated 3 times for each rat. The force was measured with a force transducer (model SM100: Interface, Scottsdale, AZ).

### Impact loading

Because we observed that the cyclic loading protocol created short spikes of high pressure followed by a plateau of relatively low pressure, the effect of pressure spikes alone was studied. This was achieved by letting a weight of 50 g fall from a height of 45 cm down onto the piston in 4 rats. To estimate the impact force, the response of the piston and fluid-filled cavity was modeled as a spring in parallel with a damper, i.e. a Kelvin solid, subjected to a step in velocity. The quotient between the damper and the spring constant is the time constant in the exponential response of such a model, and was estimated from our main experimental series to be about 0.05–0.5 s. With a spring constant of about 970 N/m, the peak impact force was estimated to be several hundred N or more. However, this estimate assumes that the piston assembly is rigidly fixed; since it was hand-held in the experiments, the peak force was probably considerably lower. This procedure was used instead of the normal loading procedure twice a day for 5 days. On the morning after the last impact loading, the rats were killed. The bone under the piston was evaluated by micro-CT, by histology, and by immunohistochemistry.

### Effect of trauma without pressure

A special device was constructed to distinguish between the effects of tissue deformation and trauma on the one hand and fluid pressure and flow on the other as causes of osteolysis. Instead of the standard piston, a similar device was inserted that did not go up and down, but which could be rotated around the axis of the piston. The device did not reach the surface of the bone, so as with the pressure piston, there was room for soft tissue formation. The surface facing the bone had a number of pegs, which became surrounded by the forming soft tissue. After soft tissue formation, the device could be rotated; the pegs would tear apart the soft tissue, thus causing trauma without adding any hydrostatic pressure. 6 rats had the trauma device inserted. After 5 days to allow formation of soft tissue, the device was rotated 20 half-turns (180°) with the same frequency as the pressure piston, for 5 days. The tissue under the piston was harvested and fixed prior to hematoxylin and eosin staining for histology.

### Statistics

The results were analyzed by one-way ANOVA and with the Tukey HSD post-hoc test (BVF, BMD, fluid velocity) and paired-samples t-test (pressure). Spearman rank correlation was used when bone volume fraction and maximum pressure and plateau pressure were analyzed.

## Results

2 animals were excluded due to technical failure of the pressure piston, one in the 14-day pressure group and one in the 5-day pressure group. 7 of 48 pressure measurements were excluded due to technical problems with pressure transducers: 2 from the 14-day pressure, 3 from the 14-day control, 1 from the 5-day pressure, and 1 from the 0-day control. One rat was excluded from the fluid pressure measurement in the bone marrow, due to leakage problems.

### In vivo fluid pressure

The pressure curves usually had a short pressure spike, followed by a constant level until the piston was unloaded and the pressure immediately decreased (Figure 3). The plateau was almost constant with minimal decrease, even during a pressure cycle of 2 min. This suggests that after the spike, no further fluid displacement occurred. Similar spikes and plateaus were seen when the pressurizing method was validated in the materials testing machine. The average duration of a fluid pressure spike was 0.28 (interquartile range 0.18–0.38) s.

**Figure 4.**
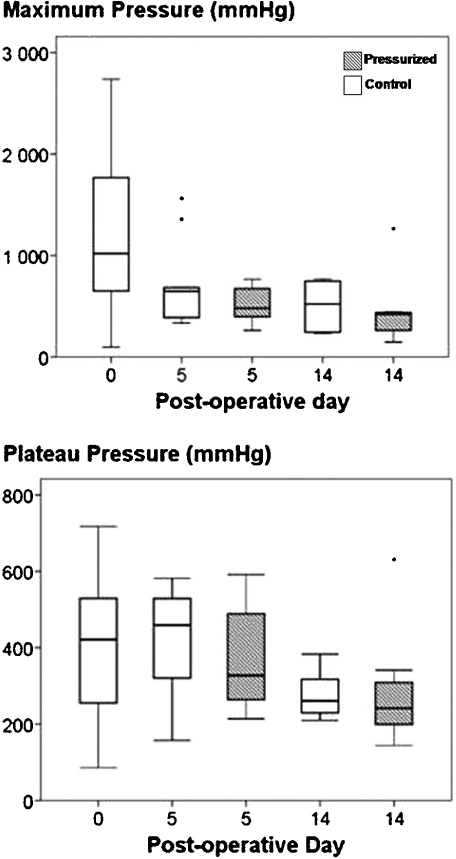
Maximum and plateau fluid pressure during the different follow-up days. Box plot with 10th, 25th, 50th, 75th and 90th percentiles.

The maximum pressure (height of the spikes) was 720 (SD 580) mmHg and the pressure plateau level was 360 (SD 150) mmHg when all groups were combined. There was no difference in maximum pressure or plateau level between pressurized animals and controls that had not been pressurized before killing (Figure 4).

There was a correlation between both maximum fluid pressure and the fluid pressure plateau on the one hand and bone volume fraction on the other: as bone was resorbed, the pressure decreased (r^2^ = 0.25, p = 0.001, and r^2^ = 0.21, p = 0.003, respectively).

### Bone morphometry and estimated fluid flow

**Figure 5.**
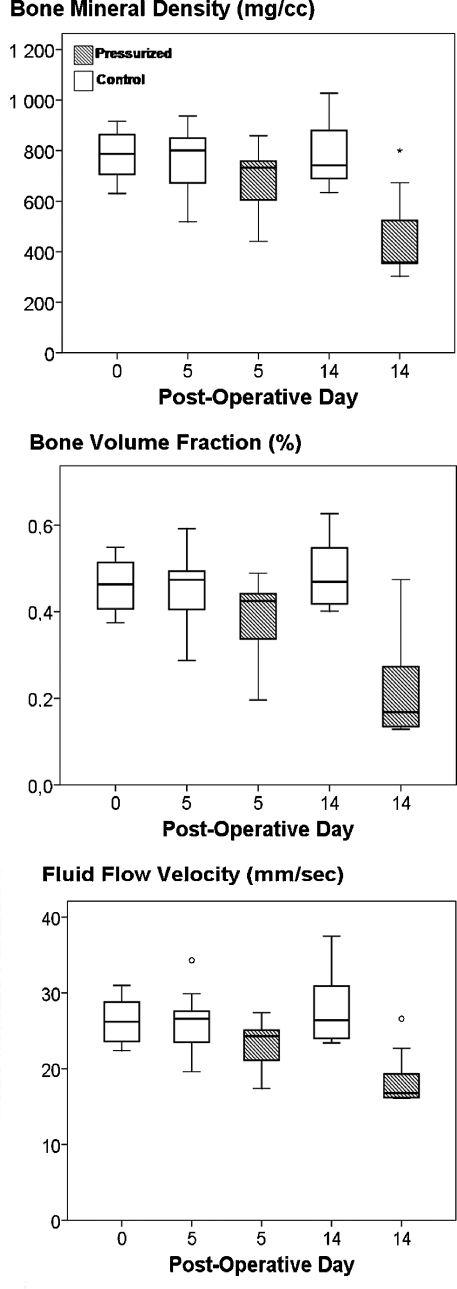
Bone density parameters from microCT and estimated fluid flow velocity. Note the decreased value in all parameters after 14 days of pressurizing. Box plot with 10th, 25th, 50th, 75th and 90th percentiles.

After 5 days of daily pressurizing, no change in bone volume fraction or bone mineral density could be seen. After 14 days, however, they had decreased by 52% and 41%, respectively, compared to the controls (Figure 5).

The estimated fluid flow velocity was 26 (SD 3) mm/s in the controls, 23 (SD 3) mm/s after 5 days of pressure, and 19 (SD 4) mm/s after 14 days of pressure (Figure 5).

### Bone morphology

**Figure 6.**
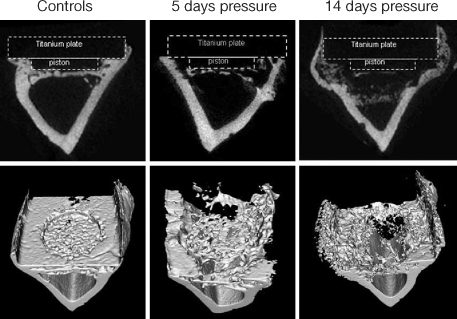
Transverse sections and 3D reconstructions of controls, 5 days pressure and 14 days pressure, showing the osteolytic development.

**Figure 7.**
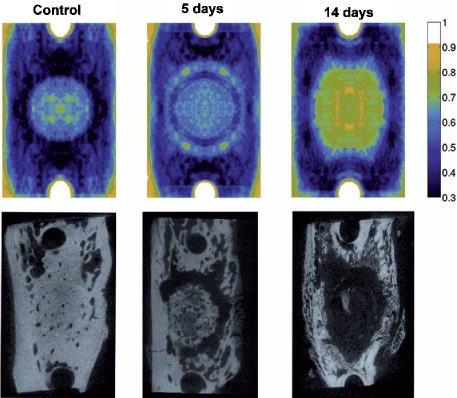
Superimposed microCT scans illustrating the average distribution of the osteolytic response (top). To produce the pictures, 9-10 scans from 9–10 different rats in each group were used and two symmetry axes imposed so that each point represent 36–40 experimental values. Here, darkest magenta represents solid bone, cyan, green and yellow represent increasing osteolysis and white represents absence of bone. Typical samples for illustration of specific bone scans below.

Non-pressurized controls showed several cavities within the cortical bone around the pressurized area at the start of the experiment. After pressurization, these cavities became enlarged and confluent, forming a ring of more or less confluent cavities around the pressurized bone area after 5 days (Figure 7). There was seldom formation of canals through cortical bone, but mostly through the more porous areas. At 14 days, the pressurized bone directly under the piston was also resorbed, leaving a large lytic cavity down to the marrow cavity, including the previously mentioned ring. Although the cortical bone adjacent to the piston was resorbed, there was no resorption of the endosteal surfaces of the marrow cavity. Instead, the lesion was separated from the marrow by reactive bone formation in the marrow, at some distance from the previous cortex (Figure 6).

### Morphology of CT scans

When micro-CT images were superimposed, the osteolytic ring formation was seen after 5 days of intermittent pressurization. It was then enlarged to an osteolytic cavity under the piston after 14 days of intermittent pressurization (Figure 7).

### Model characteristics

#### Motion-controlled loading

The results from the materials testing machine showed that the piston could be moved very rapidly without the force exceeding the manually induced level. The pressure curves showed pressure spikes that only lasted for a few tenths of a second. This means that, although the model was force-controlled for practical reasons, there was, in our standard experimental regime, an almost instantaneous fluid displacement in the tissue under the piston, and no lasting pressure was created above the plateau level of about 300 mmHg.

### Intramedullary fluid pressure

The intramedullary pressure below the piston increased by only 1 (SD 1) mmHg during pressurization. However, when a 1.2-mm hole was drilled through the surface of the bone under the piston, the pressure increased to 42 (SD 12) mmHg. The fluid pressure under the piston decreased from 1,490 (SD 1,180) mmHg to 93 (SD 16) mmHg after the hole was drilled. This means that the bone surface under the piston served as a barrier, to keep the pressure on the piston side high, and also that in the absence of such barrier function, the marrow cavity could level out the pressure.

### Fluid displacement

**Figure 8.**
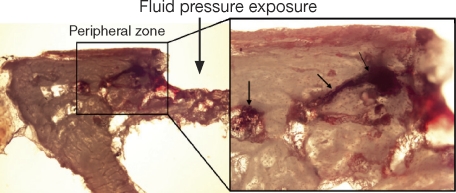
Titanium particles showing the fluid displacement during a pressure episode. Most of the titanium particles were found in cavities and canals within the bone at the peripheral zone around the pressurized area (right picture). The right picture shows titanium particles within pre-formed cavities in the peripheral zone of the bone. Arrows indicate titanium particles that were used as tracers the fluid displacement.

Titanium particles were used as tracers for fluid displacement during a 10-min pressure cycle in the controls. Most of the titanium particles, which had been displaced, were found in cavities and canals within the bone at the periphery of the pressurized area in all specimens. Only a few particles were seen in bone marrow below the pressurized bone (Figure 8).

### Impact loading

**Figure 9.**
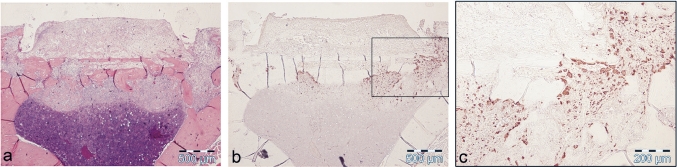
Specimen after 5 days of impact loading with different stainings. a) The soft tissue and the bone plate under the piston seen at 4× magnification (H&E), b) osteoclast activation concentrated under and beside the former piston seen at 4× magnification (immunohistochemistry for CatK), and c) activated osteoclasts within the bone plate seen at 10× magnification (CatK).

All 4 rats that were subjected to impact loading showed obvious bone resorption by histology, which was similar to that in rats exposed to the standard cyclic loading protocol (Figure 9). Multinucleate osteoclast and bone resorption lacunae were seen within the preformed cavities at the periphery, as well as in the bone plate under the piston.

### Effect of trauma without pressure

The trauma group showed intact original bone with ongoing new bone formation. The soft tissue was richly vascularized with an abundance of inflammatory cells and bleeding, confirming that the soft tissue adjacent to the bone had been traumatized. Still, none of the trauma specimens showed bone resorption.

## Discussion

Apart from the reactions to wear debris, aseptic loosening and osteolysis appear to be related to several mechanical factors such as prosthesis design, and to perioperative factors such as initial implant fixation (Robertsson et al. 2006). Fluid flow and fluid pressure have been suggested as possible mechanical initiators of loosening and osteolysis. It is unclear, however, whether the fluid pressure in itself, or the resulting fluid flow, is the crucial factor. The set of experiments presented here suggests that velocity of fluid flow is a crucial causative factor.

In our model, the spikes of fluid pressure lasted only about 0.3 s, and 20 such episodes twice a day were enough to induce osteolysis. In patients, it is possible that short loading spikes could cause moments of high flow velocity. It has been suggested that rapid motion between the stem and cement causes fluid to be ejected at high velocity through cracks in cement (Anthony et al. 1990), and similar phenomena are possible outside holes in the acetabular shell with minimal motion of the liner (Walter et al. 2004, 2005). The resorption elicited by impact loading in our model suggests the possible clinical importance of loading spikes, e.g. occurring at heel strike.

In our model, resorption occurred at pre-existing cavities within the bone, starting at the periphery around the pressurized area. This region is likely to have a high fluid flow, but somewhat less fluid pressure than the directly pressurized area. The experiment with titanium particles showed that fluid flow mostly takes this route, from the pressurized area to the periphery, and that it only finds its way through the bone underlying the pressurized area to a small degree.

The overall fluid flow velocity was estimated to be several mm/s. This is an extremely high velocity compared to what has been estimated previously for periprosthetic soft tissues (Levick 1987, Prendergast et al. 1997). The calculation is based on the observed speed of the piston and the bone volume fraction values from the micro-CT. This calculation should be relevant for the initial situation at least, when intermittent pressure treatment has started. At the later stages, when resorption cavities have formed, the available space allowing fluid flow may be greater, and therefore the velocity would be somewhat less. The magnitude of the fluid pressure was similar to that measured during different mechanical maneuvers in patients with hip prostheses, such as walking and rising from a chair (Hendrix et al. 1983).

At the start of the experiment, pressure was applied to the external bone surface, and we found no increase in fluid pressure inside the marrow cavity. After 14 days of pressure treatment, there were large osteolytic cavities extending into the bone marrow, but no resorption at the endosteal bone surfaces. Because the marrow cavity is large compared to the displacement of the piston, the fluid flow velocity inside is low. This could explain the absence of endosteal resorption around the marrow cavity. Moreover, when the resorbed bone under the piston was replaced by a seemingly dense new tissue layer of soft tissue and primitive bone, this may have protected the marrow cavity from exposure to high pressure. Even when we drilled a hole through the bone surface under the piston, the pressure in the marrow cavity was only moderately increased. This suggests that there was a dramatic difference between the soft tissue in the marrow cavity and the granulation tissue in the osteolytic lesions: the latter appears to have a much higher flow resistance, thus maintaining the pressure (Prendergast et al. 1997).

Several animal studies have shown that a certain amplitude of fluid flow or fluid pressure is needed to induce osteoclast activation (Sato et al. 1998, Qin et al. 2002). Cells respond both to deformation and to surface strain induced by fluid flow (Skerry 2006). In cell culture, a low pressure is sufficient for osteoclast activation, either via monocyte-derived macrophages or mesenchymal stem cells (Ferrier et al. 2000, Liu et al. 2009). In orthodontic tooth movements, osteoclasts are activated by a pressure of between 30 and 390 mmHg (Sato et al. 1998). In our model, trauma to the tissue adjacent to the bone did not initiate a resorptive response, as long as no pressure (and high-velocity flow) was applied. Obviously, pressure or flow induces a reaction different from the trauma-induced inflammatory reaction and apoptosis, as these can induce osteoclast activation.

In our model, reactive bone formation occurred in the soft tissue around lytic areas. Similar phenomena can be seen around loosened prostheses. This type of periprosthetic bone remodeling is unique; it is not seen under normal conditions and may not be apparent in other pathological conditions (Takagi et al. 2001). It is a high-turnover bone remodeling with poor bone quality.

The ability of short bursts of high velocity of fluid flow to induce osteolytic bone lesions may be important. Better knowledge in this field might allow new means of avoiding osteolysis around orthopedic prostheses through changes in design or changes in insertion technique.
